# Case report: Acute pancreatitis in lung adenocarcinoma with small cell transformation after multiple line targeted therapy

**DOI:** 10.3389/fonc.2024.1274034

**Published:** 2024-01-19

**Authors:** Yaoyao Jing, Xiaoxiao Li, Xiaoyuan Sun, Minghan Ren, Ruoxi Xiao, Jiayu Zhao, Zimin Liu

**Affiliations:** ^1^ Department of Medcine, Qingdao University, Qingdao, China; ^2^ Department of Digestive Oncology, The Affiliated Hospital of Qingdao University, Qingdao, China

**Keywords:** pancreatic metastasis, lung adenocarcinoma, small cell lung cancer transformation, metastasis-induced acute pancreatitis, case report

## Abstract

In lung cancer, metastasis to the liver, bones, brain, and adrenal glands is more commonly observed, whereas pancreatic metastasis from lung cancer is relatively rare. We present a case of a patient with an 8-year history of lung adenocarcinoma (LUAD) who was admitted to our institution exhibiting symptoms consistent with acute pancreatitis. Subsequent histopathological examination through puncture confirmed the occurrence of pancreatic metastasis originating from small cell lung cancer (SCLC). During a multidisciplinary team discussion, we reached a consensus in diagnosing the patient with post-transformation small cell carcinoma alongside moderately severe pancreatitis, which was determined to be a consequence of pancreatic metastasis. The patient received a regimen of etoposide and cisplatin chemotherapy. This unique clinical case highlights the importance of further investigating the factors contributing to pancreatic metastasis in patients with lung cancer, as the underlying mechanisms remain unclear. Understanding these exceptional metastatic events is vital in devising effective therapeutic strategies and improving patient prognosis. Our findings emphasize the need for continued surveillance and comprehensive management of lung cancer patients, particularly those with resistant forms of the disease, to promptly identify and address the progression of metastatic events to uncommon sites such as the pancreas.

## 1 Introduction

Pancreatic metastases are rare and mostly discovered during the autopsy (1). Among cases of metastasis, renal cell carcinoma is the most common primary tumor (38.4%), followed by lung cancer (24.5%), colorectal cancer (11.3%), and sarcoma (6.3%) (2). In the context of lung cancer, intra-abdominal metastases typically involve the liver and adrenal gland through vascular routes, while pancreatic metastasis is uncommon. Particularly rare is the occurrence of pancreatic metastasis originating from non-small cell lung cancer (NSCLC) that undergoes a transformation into small cell lung cancer (SCLC). In this report, we present a noteworthy case of such a patient who was admitted under our care.

## 2 Case report

**Figure 1:**
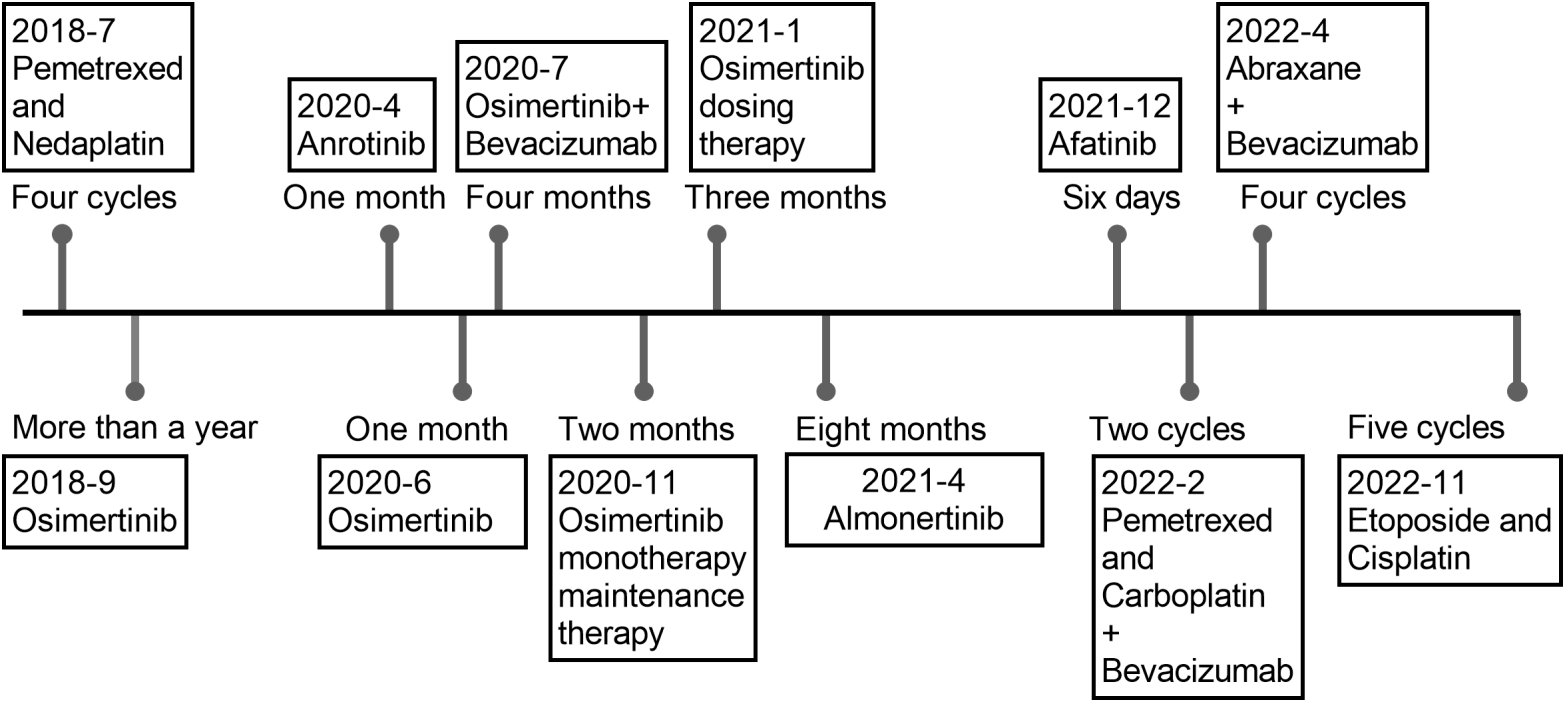
Chemotherapy and targeted therapy for advanced lung adenocarcinoma.

**Figure 2:**
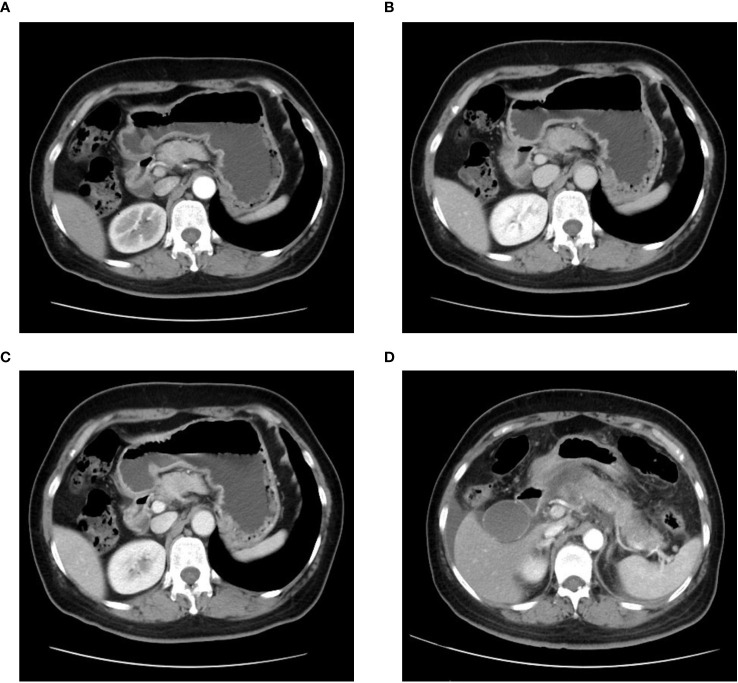
Computed tomography (CT) of the epigastric region showing a pancreatic mass with indeterminate nature is depicted in **(A)** arterial phase, **(B)** venous phase, and **(C)** delayed phase. **(D)** An enhanced CT image of the epigastric region taken during the patient**’**s initial episode of pancreatitis.

**Figure 3:**
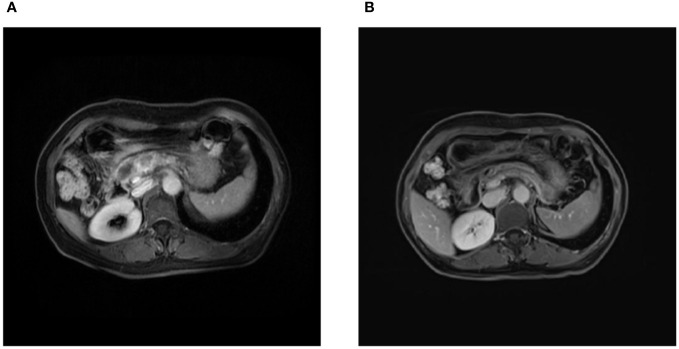
Magnetic resonance imaging findings. **(A)** Initial magnetic resonance imaging on admission revealed two masses in the neck and leptomeninges of the pancreas, respectively, along with an encapsulated exudate in the head of the pancreas. **(B)** Follow-up magnetic resonance imaging after 3 cycles of chemotherapy demonstrated a reduction in the size of the pancreatic masses.

**Figure 4:**
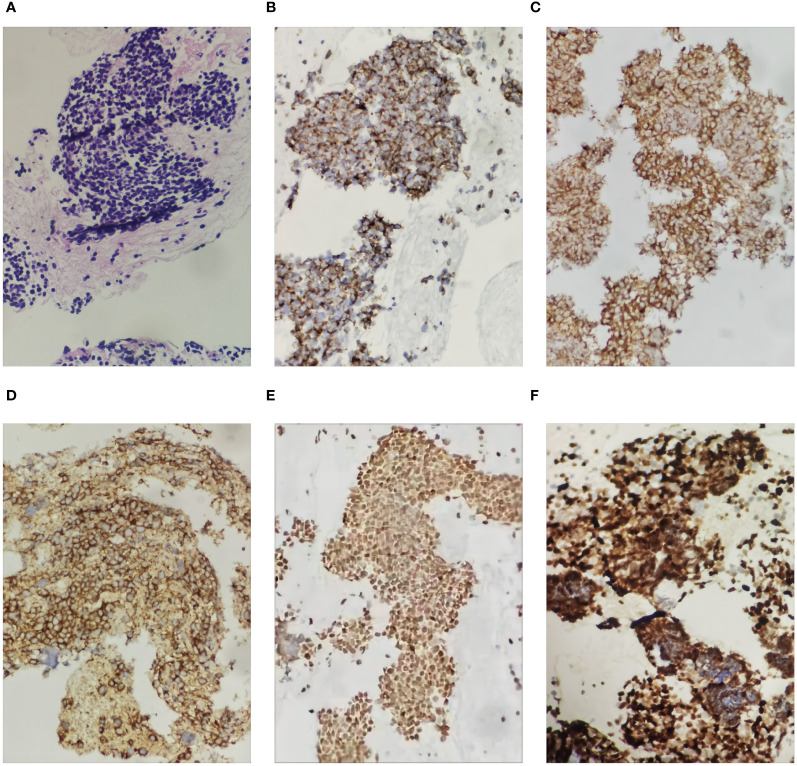
Histological images of the pancreatic tumor showing small cell carcinoma in the pancreas (hematoxylin and eosin staining (HE), ×200 magnification). The small cell carcinoma was positive with immunostaining for CK, Syn, CD56, TTF-1 and Ki67 (200X): **(A)** was HE staining of the specimen, **(B)** was immunohistochemical staining with CK, **(C)** was immunohistochemical staining with Syn, **(D)** was immunohistochemical staining with CD56, **(E)** was immunohistochemical staining with TTF-1, and **(F)** was immunohistochemical staining with Ki67.

A 52-year-old female patient presented with epigastric pain and persistent colic in the left abdominal region. The patient had a history of invasive adenocarcinoma in the right upper lobe of the lung, for which she underwent right upper lobe resection and received adjuvant chemotherapy with pemetrexed and carboplatin eight years prior to admission. Genetic testing revealed the presence of an epidermal growth factor receptor (EGFR) exon 21 mutation, resulting in a 3.5-year administration of maintenance therapy with erlotinib-targeted drugs. However, a recurrence of the tumor was detected by positron emission tomography-computed tomography (PET-CT) scans, necessitating a right lobectomy followed by postoperative chemotherapy with pemetrexed and nedaplatin. Postoperative genetic analysis identified EGFR exon 21 L858R mutation and EGFR exon 20 T790M mutation. Subsequently, the patient underwent two years of oral osimertinib-targeted therapy following the completion of chemotherapy. Nonetheless, in April 2020, disease progression was identified based on elevated carcinoembryonic antigen (CEA) levels observed during a reexamination. Consequently, the patient’s treatment was switched to anrotinib, albeit discontinued after only one month due to the occurrence of adverse effects. As a result, oral osimertinib was reintroduced as the choice of therapy, yet CEA levels remained persistently elevated during subsequent evaluations. In July 2020, a combination therapy comprising osimertinib and bevacizumab was administered, resulting in a significant reduction in CEA levels. However, after a duration of four months, the administration of bevacizumab was terminated due to drug-induced liver injury, and the patient continued with maintenance therapy utilizing oral osimertinib alone. Unfortunately, CEA monitoring in January 2021 unveiled a subsequent increase, prompting an escalation in the osimertinib dosage. In April 2021, CEA levels persistently rose without any signs of improvement, leading to the decision to switch to almonertinib as the oral drug. During a physical examination in December 2021, the patient was diagnosed with cervical lymphadenopathy, confirmed by needle biopsy to be metastatic carcinoma, while whole-body bone imaging showed no irregularities. Consequently, the oral drug was switched to Afatinib. However, this medication had to be discontinued a week later due to intolerable side effects, including oral mucositis, weight loss, and diarrhea. Moreover, additional genetic testing revealed the presence of TP53, MET, ROS1, and ALK mutations (**Table 1**), indicating the existence of additional drug resistance gene mutations. In January 2022, computed tomography (CT) scans revealed further enlargement of the cervical lymph nodes. The patient was hospitalized for a comprehensive treatment approach consisting of chemotherapy using pemetrexed and carboplatin, alongside targeted therapy employing bevacizumab. In addition, supraclavicular radiotherapy was administered as part of the therapeutic regimen. Regrettably, after two cycles of chemotherapy, bone metastases were detected, indicating disease progression. Thus, the therapy regimen was modified to include abraxane and bevacizumab (The course of chemotherapy and targeted therapy after recurrence is shown in **Figure 1**). During hospitalization for chemotherapy, an enhanced CT scan of the upper abdomen showed patchy low-density shadows in the head of the pancreas, measuring approximately 19 mm × 17 mm (**Figure 2**). The degree of enhancement observed during the scan was lower than that of normal pancreatic tissue, and the nature of the shadows remained undetermined. One month later, the patient underwent a CT scan due to abdominal pain, which revealed patchy low-density shadows in the pancreatic head area with indistinct borders (**Figure 2**). These findings were suggestive of acute pancreatitis. Further enzymatic screening confirmed the presence of pancreatitis, with elevated levels of amylase (969.10 U/L; normal range: 30-110 U/L) and lipase (6092.00 U/L; normal range: 23-300 U/L). Tumor marker analysis indicated elevated levels of pro-gastrin releasing-peptide (pro-GRP) (173.69 pg/mL; normal range: 0-63 pg/mL) and CEA (39.60 ng/mL; normal range: 0-5 ng/mL). Magnetic resonance imaging (MRI) indicated mildly enhancing masses measuring approximately 20 mm × 13 mm and 13 mm × 10 mm in the neck and uncinate process of the pancreas, respectively, along with encapsulated effusion in the pancreatic head (**Figure 3**). Furthermore, an ultrasound-guided gastroscopy was performed for cytopathology examination, revealing a small cell carcinoma lesion in the pancreatic head that tested positive for CK, CD56, Ki67, Syn, and TTF-1 (**Figure 4**). Based on the patient’s clinical history and pathological findings, metastatic tumors originating from the lung were highly suspected.

**Table 1:** Gene mutation profile.

Test date	Biologically Relevant Genomic Variants	gene subregion	Variant Allele Fraction
March 30, 2015	*EGFR*	Exon 21	–
June 6, 2018	*EGFR p.L858R*	Exon 21	–
	*EGFR p.T790M*	Exon 20	–
December 9, 2021	*EGFR p.L858R*	Exon 21	31.41%
	*TP53 p.E271*	Exon 8	95.07%
	*MET p.I1345T*	Exon 21E	66.61%
	*ROS1 p.D2213E*	Exon 42	29.31%
	*ALK p.R1231P*	Exon 24	26.98%

"-" represents no detection for variant allele fraction.

At a multidisciplinary team discussion, we finally diagnosed SCLC transformation triggered by resistance to targeted therapy for lung adenocarcinoma (LUAD), accompanied by pancreatic metastasis arising from the transformed SCLC and concomitant moderate to severe pancreatitis.

The patient was managed for acute pancreatitis with a treatment regimen consisting of a somatostatin analog, a protease inhibitor, acid suppression, and analgesia. Following the resolution of pancreatitis, she received chemotherapy with etoposide and cisplatin. Following three cycles of chemotherapy, MRI findings indicated a reduction in the size of the pancreatic lesions (**Figure 3**), and her treatment response was evaluated as stable disease (SD) according to the Response Evaluation Criteria in Solid Tumors (RECIST) scoring criteria. Unfortunately, within a span of seven months from the initial diagnosis of pancreatic metastasis, the patient developed brain metastasis accompanied by a brain hemorrhage.

## 3 Discussion

Pancreatic metastases are relatively uncommon in living individuals. Autopsy studies have shown that the incidence of pancreatic secondary tumors is around 15% in cases of malignant tumors (1). In a retrospective analysis conducted at the Peking Union Medical College Hospital, the incidence of pancreatic metastasis from lung cancer was found to be approximately 0.25% (42/17,045) (3). Among the 42 patients, it was observed that SCLC constituted 43% of cases, while NSCLC accounted for 57%, wherein adenocarcinoma was the prevailing subtype, contradicting the previous notion of SCLC predominance (4). This observation may be attributed to the considerable advancements in the treatment of NSCLC, including targeted therapy and immunotherapy, resulting in prolonged patient survival and, consequently, an increased occurrence of distant organ metastasis during disease progression. Currently, epidermal growth factor receptor tyrosine kinase inhibitors (EGFR-TKIs) as monotherapy is the widely recommended first-line treatment for patients with advanced NSCLC harboring EGFR mutations (5). Third-generation EGFR-TKIs, such as Osimertinib, have also been introduced and show promise (6). Despite their high potency, the development of acquired resistance to these drugs is inevitable. Resistance mechanisms to third-generation EGFR-TKIs can be classified into on-target resistance and off-target resistance, particularly EGFR-dependent resistance mechanisms, such as activation of bypass signaling, oncogenic fusions, alterations in downstream pathways, histologic transformations, and alterations in cell cycle genes (7, 8).

The mechanisms underlying the phenotypic transformation of SCLC following treatment with EGFR-TKIs are still unclear. Currently, SCLC transformation is primarily observed in patients with EGFR mutant NSCLC, representing the most significant source of such conversion (9). Previous studies have suggested that this transformation process may require prolonged exposure to TKIs, with a median duration of approximately 19 months (range 1-61 months) (10). Furthermore, it has been noted that patients who derive long-term benefits from these agents face an elevated risk of transdifferentiating to the SCLC phenotype upon disease progression (11). The ability of NSCLC to transform into SCLC likely arises from their shared cellular origin. LUADs with EGFR mutations originating from alveolar type II cells (12) may undergo transdifferentiation into SCLC under the selective pressures exerted by TKIs therapy (13). It has been shown that the assessment of RB1 and TP53 status in LUADs treated with EGFR-TKIs is essential for predicting the occurrence of small cell transformation. Molecular fingerprinting and immunohistochemical comparisons were performed before and after the neuroendocrine transformation, revealing the detection of TP53 and RB1 loss and inactivation prior to the transformation (14). While RB1 inactivation has been deemed necessary, it is not sufficient for the development of SCLC. Researchers have found that ASCL1 overexpression may lead to RB1 phosphorylation and inactivation by inducing cyclin-dependent kinase 5 (CDK5), and that RB1 inactivation induces proliferation and possible transformation of TP53 mutant cells (15).

SCLC transformation may occur during targeted therapy for NSCLC, posing a significant challenge in patient management. Distinguished by its propensity for lymphatic and hematogenous metastasis, SCLC’s transformed components can give rise to distant metastases, including the pancreas. The clinical manifestations of metastatic pancreatic tumors can be asymptomatic or non-specific, such as epigastric pain, vomiting, nausea, and weight loss. Approximately 43% of patients may experience symptoms specifically related to pancreatic metastasis, such as low back pain, acute pancreatitis, and obstructive jaundice (16). Metastasis-induced acute pancreatitis (MIAP) frequently occurs during the advanced stages of lung cancer and can also manifest as an initial symptom of the disease (17). Additionally, there are notable differences between tumor-induced acute pancreatitis and non-tumor-induced acute pancreatitis. Lung cancer patients with MIAP tend to be older, with higher rates of primary pancreatic duct dilatation and abdominal lymphadenopathy. They also exhibit lower levels of hemoglobin and hematocrit, which serve as diagnostic clues warranting clinical attention (18). Therefore, unexplained acute pancreatitis in an older individual should raise suspicion for this condition (17).

Serological tests have demonstrated the potential to predict early conversion of adenocarcinoma to SCLC. Notably, serum proGRP has emerged as a promising clinical tumor marker for SCLC (19, 20). It exhibits a superior sensitivity of approximately 75% and specificity of around 90% for SCLC, surpassing that of neuron-specific enolase (NSE) (21–23).

The gold standard for diagnosing pancreatic metastasis from lung cancer is through pathological histological or cytological examination of pancreatic masses. Endoscopic ultrasound-guided fine-needle aspiration (EUS-FNA) is a safe and minimally invasive method used for the diagnosis of non-primary pancreatic neoplasms (24). It is worth noting that secondary tumors account for about 4% of pancreatic specimens, and approximately one-third of them are clinically misdiagnosed as primary tumors of the pancreas (25). Secondary pancreatic tumors not only have a low incidence but also a high rate of misdiagnosis. Therefore, metastatic tumors tumors should be entertained in both the clinical and pathological differential diagnosis of pancreatic neoplasia. Furthermore, the prompt utilization of Next-Generation Sequencing (NGS) during the initial phases of the disease holds the potential to facilitate the identification of a pancreatic mass (26).

Treatment strategies for SCLC and NSCLC differ significantly. It is crucial to recognize the small cell component in clinical practice since patients with SCLC have higher response rates to platinum-etoposide and taxanes after transformation (27). Previous studies have reported that chemotherapy with cisplatin and irinotecan can not only achieve remission of pancreatitis in cases of pancreatic metastasis of SCLC, but also lead to significant regression of metastatic lesions (28, 29). Serum ProGRP levels have been shown to have an excellent correlation with treatment response in SCLC patients, making it a useful marker for monitoring treatment effectiveness and prognosis (19, 30). In this case, the patient underwent chemotherapy with etoposide and cisplatin regimen. After three cycles of treatment, the mass was assessed to have shrunken; however, the patient was later admitted to the internal medicine department for pancreatitis, which interrupted the fifth cycle of chemotherapy. This scenario highlights the potential benefits of chemotherapy against the small cell component of tumors and underscores the importance of patients adhering to the treatment protocol and receiving timely chemotherapy to avoid possible interruptions that can worsen their condition. The limitation of this study is the interruption of the chemotherapy regimen assessment, which prevented the observation of potential survival improvements from continuous etoposide and cisplatin regimen chemotherapy.

MIAP primarily occurs due to tumor deposition or extrinsic compression of metastatic lymph nodes, resulting in ductal obstruction (31). It is crucial to consider Acute obstructive suppurative pancreatic ductitis (AOSPD) and obstructive pancreatitis in patients with metastatic pancreatitis who experience severe abdominal pain and inflammation. APSOD is defined as a bacterial infection of the pancreatic duct, leading to duct obstruction, purulent pancreatic juice discharge, or positive pancreatic juice cultures (32). Pancreatic duct drainage, particularly the utilization of nasopancreatic duct drainage (NPD), has demonstrated significant advantages in effectively managing AOSPD (33). Treating MIAP is a complex process that necessitates prompt administration of chemotherapy regimens. Additionally, medical drug therapy during pancreatitis attacks and pancreatic duct drainage, when deemed necessary, should also be considered. Therefore, it is crucial to establish interdisciplinary diagnostic and treatment protocols to ensure comprehensive management of this condition.

Pancreatic metastasis in lung cancer patients is indicative of stage IV disease, often accompanied by concurrent metastases to the bone, brain, and adrenal glands (34). Multivariate analysis has revealed that lung cancer patients with MIAP have a poor prognosis, with a median overall survival (OS) of 8.8 months (3). Therefore, early detection and accurate differentiation of pancreatic metastasis hold significant clinical significance.

In conclusion, this case presents a rare occurrence of pancreatic metastasis subsequent to small cell transformation in LUAD, which poses considerable diagnostic challenges and is prone to misdiagnosis. Hence, clinicians should exercise vigilance regarding the potential for metastases when managing pancreatic tumors. The utilization of tumor biopsy and NGS is recommended for precise identification. However, there currently exists a dearth of standardized clinical criteria for the diagnosis and treatment of pancreatic metastasis following small cell transformation in LUAD. Several unanswered questions persist, including the formulation of appropriate treatment regimens, the potential for distinct biological behavior in transformed SCLC, and the potential necessity of novel treatment strategies. Further clinical research is necessary to address these uncertainties.

## Data availability statement

The raw data supporting the conclusions of this article will be made available by the authors, without undue reservation.

## Ethics statement

Written informed consent was obtained from the individual(s) for the publication of any potentially identifiable images or data included in this article.

## Author contributions

YJ: Writing – original draft, Data curation, Investigation, Methodology, Visualization, Writing – review & editing. XL: Conceptualization, Resources, Writing – review & editing. XS: Supervision, Writing – review & editing. MR: Writing – review & editing. RX: Visualization, Writing – review & editing. JZ: Writing – review & editing. ZL: Conceptualization, Supervision, Writing – review & editing.
